# Adaptive Nonlinearity Compensation System for Integrated Temperature and Moisture Sensor

**DOI:** 10.3390/mi10120878

**Published:** 2019-12-13

**Authors:** Guohong Chen, Shengjun Zhou, Jie Ni, Hao Huang

**Affiliations:** 1 School of Information & Electrical Engineering, Zhejiang University City College, 51 Huzhou Street, Hangzhou 310015, China; chenguohong@zucc.edu.cn (G.C.);; 2 Zhejiang Academy of Agricultural Sciences, 198 Shiqiao Road, Hangzhou 310021, China; 3 Hubei Key Lab of Ferro- & Piezoelectric Materials and Devices, Faculty of Physics and Electronic Science, Hubei University, 368 Youyi Street, Wuhan 430062, China; 4 Key Laboratory of Wireless Sensor Network & Communication, Shanghai Institute of Microsystem and Information Technology, Chinese Academy of Sciences, 865 Changning Road, Shanghai 200050, China

**Keywords:** temperature and moisture sensor, adaptive order regulating, temperature-dependent nonlinearity, nonlinearity compensation

## Abstract

Measuring temperature and moisture are important in many scenarios. It has been verified that temperature greatly affects the accuracy of moisture sensing. Moisture sensing performance would suffer without temperature calibrations. This paper introduces a nonlinearity compensation technique for temperature-dependent nonlinearity calibration of moisture sensors, which is based on an adaptive nonlinear order regulating model. An adaptive algorithm is designed to automatically find the optimal order number, which was subsequently applied in a nonlinear mathematical model to compensate for the temperature effects and improve the moisture measurement accuracy. The integrated temperature and moisture sensor with the proposed adaptive nonlinear order regulating nonlinearity compensation technique is found to be more effective and yield better sensing performance.

## 1. Introduction

**Figure 1:**
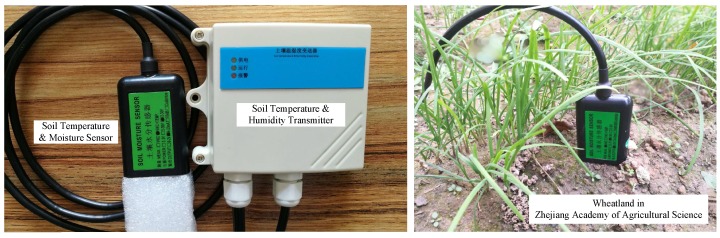
Temperature and moisture sensor deployed in the wheatland.

Temperature and moisture sensors have extensive applications in many industries, including agriculture, food, pharmaceuticals, mining, construction, and so on. For instance, integrated temperature and moisture sensors have been widely deployed in the wheatland in Zhejiang academy of agricultural sciences, as shown in Figure 1.

Many kinds of moisture sensors have been researched and developed. However, previous researchers have proven that temperature variation has a significant and nonlinear influence on moisture measurement results [1,2]. On the one hand, the materials which make up the moisture sensing elements behave in a nonlinear way with the temperature. On the other hand, all moisture sensors must contain active electronics, which are characterized by a nonlinear relationship with temperature [3,4]. To obtain a correct moisture reading, primitive moisture measurement data need to be adjusted according to temperature, often in a nonlinear manner [3,4,5,6].

In general, a commercial sensor would be made of a temperature sensing unit and a moisture sensing unit integrated together. Conventionally, moisture adjustment by temperature was achieved with look-up tables (LUTs), which are large datasets consisting of measured temperatures, measured moistures, and corresponding adjusted moisture values [6]. Manufacturers perform huge numbers of tests under various temperatures and moisture levels in order to construct LUTs. However, the accuracy of this nonlinear mapping relationship depends on the precision of the measurements and the size of the LUT [7,8]. LUT-based adjustment can be highly accurate; however, a sensor unit can potentially be used in a huge range of temperatures, thus requiring a huge LUT to accurately provide moisture level readings. Despite this, the LUT method is effective and straightforward, although its huge size means it cannot be implemented in a microcontroller with limited memory.

A mathematical model of nonlinear adjustment can overcome the shortcomings of conventional sensors, and to a large extent, reflect and compensate the complex effect of temperature on moisture readings [9,10,11]. However, the existing compensation methods often use nonlinear mathematical models with fixed orders, which may not reach optimal error under different usage environments, thus making it difficult to attain the best compensation effects. In addition, these models are hard to migrate from one type of sensor to another.

In order to achieve a better sensing performance and lower measurement error, we propose a nonlinear compensation method for adaptive order adjustment. A high-order mathematical formula is used to reflect the nonlinear relationship between the actual moisture and the sensor’s measured probe voltage [12,13]. A high-order mathematical compensation model with the smallest error is selected by an adaptive order selection module. The experimental results verify that the measurement accuracy of this adaptive order nonlinearity compensated temperature and moisture sensor is significantly improved.

## 2. System Construction

**Figure 2:**
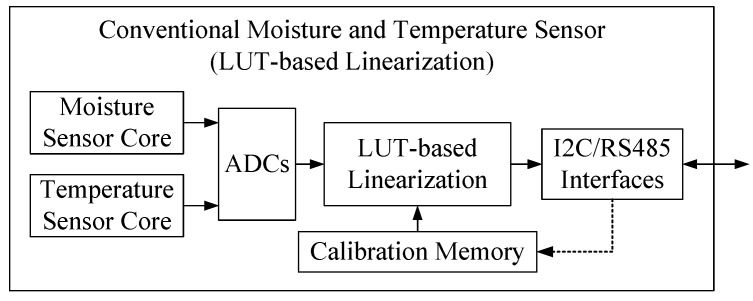
System diagram of the conventional moisture and temperature sensor.

The structure of a conventional temperature and moisture sensor is shown in Figure 2. A temperature sensor and a moisture sensor are integrated in the system. The analog measurement results are digitized by an analog-to-digital converter (ADC) and then calibrated by a LUT-based linearization module [14]. The calibration memory should be large enough to store a large LUT for high-accuracy temperature and moisture sensing, thus this traditional method requires a large storage capacity.

**Figure 3:**
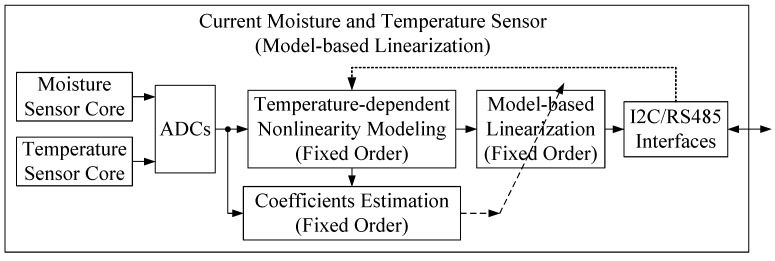
Architecture of the current sensor with model-based temperature-dependent nonlinearity compensation system.

As shown in Figure 3, the temperature-moisture sensor uses a fixed-order temperature-dependent nonlinear compensation model rather than a look-up table. Temperature-dependent nonlinear modeling requires three sets of data, including internally detected moisture, internally detected temperature, and true moisture transmitted from the outside via the RS485 interface [15]. When determining the fixed-order nonlinear model, the least square (LS) algorithm is used to estimate the model coefficients.

**Figure 4:**
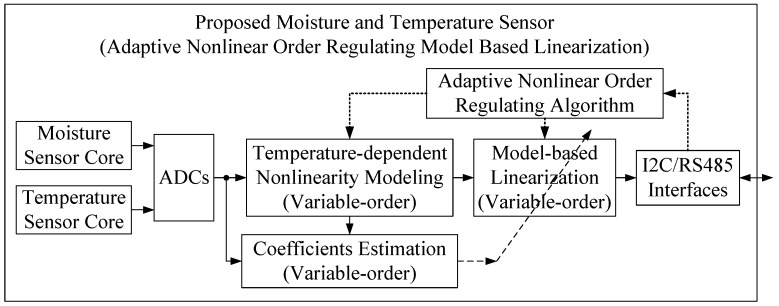
System architecture of the proposed temperature and moisture sensor with adaptive nonlinear order regulating model-based linearization.

The adaptive order-adjusted temperature and moisture sensor proposed in this paper, as shown in Figure 4, adds an adaptive order adjustment and feedback module based on the Figure 3 fixed-order nonlinear compensation model. The same as the architecture shown in Figure 3, temperature-dependent nonlinear modeling requires the same three sets of data, including internally detected temperature, internally detected moisture, and true moisture. The adaptive nonlinear order regulation algorithm module is added to search for the model with the optimal nonlinear order and the best nonlinearity compensation performance. During the searching process, the LS algorithm is used to estimate coefficients of the variable order models.

The advantages and disadvantages and degree of difficulty of the three temperature and moisture sensors are shown in Table 1. All of the three integrated sensors can be implemented by a common sensor with temperature and moisture sensor cores and an STM32L4R5 microcontroller with 2048 KB flash, 640 KB ram, 12-bit DACs (digital-analog convertor), and 16-bit ADCs (analog-digital convertor) from STMicroelectronics in Geneva, Switzerland.

## 3. Adaptive Nonlinear Order Regulating Model-Based Nonlinearity Compensation Algorithm

We performed a total of 12 sets of tests among an adaptively-ordered nonlinearly-corrected sensor, a noncalibrated sensor, and a fixed third-order nonlinearly-corrected sensor just as in [15]. The true moisture levels of each test were fixed, and moisture was measured in a changing environment from $5{\;}^{\circ }$C to $40{\;}^{\circ }$C, and subsequently compared with the true moisture values. The nonlinear effects of temperature on moisture measurements are clearly observed. The nonlinear modeling and the coefficients estimation are brought out by the experimental data of moisture sensing, as listed in Table 2. To be clear, the true moisture values in the Table 2 were measured using the weighting method after drying, which is a well-known standard method of moisture measurement [16,17].

The empirical Topp’s equation describes the nonlinear relationship between the volumetric water content θ and the dielectric constant of water $\varepsilon_{b}$ [18]:

(1) $$\begin{array}{c} \theta =-5.3\times 10^{-2}+2.92\times 10^{-2}\varepsilon_{b}-5.5\times 10^{-4}{\varepsilon_{b}}^{2}+4.3\times 10^{-6}{\varepsilon_{b}}^{3}. \end{array}$$

Thus, during the measurement, we expected the measured moisture $\theta_{meas}$ to vary with temperature according to the following relationship:

(2) $$\begin{array}{cc} \theta_{meas}\left(i,T_{m}\right)= & -5.3\times 10^{-2}+2.92\times 10^{-2}\varepsilon_{b,meas}\left(i,T_{m}\right)-5.5\times 10^{-4}{\varepsilon_{b,meas}}^{2}\left(i,T_{m}\right)\\ \; & +4.3\times 10^{-6}{\varepsilon_{b,meas}}^{3}\left(i,T_{m}\right), \end{array}$$

where i=1,2,…,12, and $T_{m}=5,10,\ldots ,40{\;}^{\circ }$C, and where m=1,2,…,8.

During the test, the dielectric constant of water decreases with increasing temperature [19,20,21], and so a temperature-dependent nonlinear model needs to be constructed to compensate for the effects of temperature [15,22,23]. The relationship between the fixed actual moisture value $\theta_{real}$ and the actual water dielectric constant can be seen in Equation (3). The nonlinear relationship we constructed can be seen in Equations () and ().

(3) $$\begin{array}{cc} \theta_{real}\left(i\right)= & -5.3\times 10^{-2}+2.92\times 10^{-2}\varepsilon_{b,real}\left(i\right)-5.5\times 10^{-4}{\varepsilon_{b,real}\left(i\right)}^{2}+4.3\times 10^{-6}{\varepsilon_{b,real}\left(i\right)}^{3} \end{array}$$

(4) $$\begin{array}{cc} \varepsilon_{b,real}\left(i\right)= & \varepsilon_{b,meas}\left(i,T_{m}\right)\cdot C_{n}\left(i,T_{m}\right) \end{array}$$

(5) $$\begin{array}{cc} C_{n}\left(i,T_{m}\right)= & 1-a_{1}\left(i\right)\times \left(T_{m}-25\right)-a_{2}\left(i\right)\times {\left(T_{m}-25\right)}^{2}-\ldots -a_{n}\left(i\right)\times {\left(T_{m}-25\right)}^{n}, \end{array}$$

where $\theta_{real}\left(i\right)$ is the real moisture of the $i_{th}$ test; $\varepsilon_{b,real}\left(i\right)$ is the real dielectric permittivity of water in general; $C_{n}\left(i,T_{m}\right)$ is temperature-related nonlinear factor; and $a_{k}\left(i\right),k=1,2,\ldots ,n$ are the *n* nonlinear coefficients to be estimated.

Estimated nonlinear coefficients are applied to validate the performance of the temperature-related nonlinearity compensation. By plugging ${\hat{a}}_{k}\left(i\right),k=1,2,\ldots ,n$ and $\varepsilon_{b,meas}\left(i,T_{m}\right)$ into Equations (3)–(), one gets the estimation of the real moisture values ${\hat{\theta }}_{real}\left(i\right)$. For each test *i*, the moisture error ratios with and without nonlinearity compensation [15], expressed by $R_{e,w}\left(i\right)$ and $R_{e,wo}\left(i\right)$, are respectively defined as

(6) $$\begin{array}{cc} R_{e,w}\left(i\right)= & \sum_{m=1}^{M}\left|1-\frac{{\hat{\theta }}_{i,nl}}{\theta_{i,nl}}\right|\times \frac{1}{M}\times 100 \end{array}$$

(7) $$\begin{array}{cc} R_{e,wo}\left(i\right)= & \sum_{m=1}^{M}\left|1-\frac{\theta_{i,meas}}{\theta_{i,nl}}\right|\times \frac{1}{M}\times 100\; \end{array}$$

where *m* is the temperature index, and *M* is the total number of the temperatures.

The specific calculation process can be seen in Algorithm 1:
**Algorithm 1** Calculation process.**Require:** $\theta_{real}\left(i\right)$: real moisture of the $i_{th}$ test;$\varepsilon_{b,real}\left(i\right)$: real dielectric permittivity of water;$C_{n}\left(i,T_{m}\right)$: temperature-related nonlinear factor;$a_{k}\left(i\right),k=1,2,\ldots ,n$:the nonlinear coefficients to be estimated;*n*: Nonlinear correction equation order;**Ensure:** *P*:the optimal corrected nonlinear order;1:initial n=0; $R_{e,w,tmp}=0$2:**repeat**3:    n=n+1;4:    compute $\varepsilon_{b,meas}\left(i,T_{m}\right)$ by solving Equation (2) with the measured moisture $\theta_{meas}\left(i,T_{m}\right)$ in each row of Table 2;5:    acquire $\varepsilon_{b,real}\left(i\right)$ by solving Equation (3) with the real moisture $\theta_{real}\left(i\right)$ in each row of Table 2;6:    obtain $C_{n}\left(i,T_{m}\right)$ by plugging $\varepsilon_{b,meas}\left(i,T_{m}\right)$ and $\varepsilon_{b,real}\left(i\right)$ into Equation (4);7:    compute $a_{k}\left(i\right),k=1,2,\ldots ,n$ by solving Equation (5) by LS algorithm;8:    acquire $R_{e,wo}\left(i\right)$;9:    obtain $R_{e,w}\left(i\right)$;10:    determine $S\left(i\right)=boolean(mean\left(R_{e,w}\left(i\right)\right)>R_{e,w,tmp})$;11:    **if** (S(i)==0) **then**12:        set $R_{e,w,tmp}=mean\left(R_{e,w}\left(i\right)\right)$13:        set P=n14:    **else**15:        set P=n−116:    **end if**17:**until** (S(i)==1)18:$\min R_{e,w}\left(i\right)$ is found, *P* is the optimal corrected nonlinear order.

In particular, the LS algorithm in Step 4 can be represented in matrix form as follows:

For the *i*th moisture index, Equation (4) can be rewritten in the matrix form as

(8) $$C_{i}=\mathrm{ones}\left(M,1\right)-T\cdot a_{i},$$

where the matrixes with *n*th nonlinear order are

$$\begin{array}{cc} C_{i}= & \;{\left[\;C\left(i,T_{1}\right),\;C\left(i,T_{2}\right),\;\ldots ,\;C\left(i,T_{M}\right)\;\right]}^{T}\\ \mathrm{ones}(M,1)= & \;{\left[\;1,\;1,\;1,\;\ldots ,\;1\;\right]}^{T}\\ T= & \;[\;\left(T_{1}-25\right),\;{\left(T_{1}-25\right)}^{2},\;{\left(T_{1}-25\right)}^{3},\;\ldots ,\;{\left(T_{1}-25\right)}^{n};\\ \; & \;\;\;\left(T_{2}-25\right),\;{\left(T_{2}-25\right)}^{2},\;{\left(T_{2}-25\right)}^{3},\;\ldots ,\;{\left(T_{2}-25\right)}^{n}\\ \; & \;\;\;\ddots \\ \; & \;\;\;\left(T_{M}-25\right),\;{\left(T_{M}-25\right)}^{2},\;{\left(T_{M}-25\right)}^{3},\;\ldots ,\;{\left(T_{M}-25\right)}^{n}]\\ a_{i}= & \;{\left[\;a_{1}\left(i\right),\;a_{2}\left(i\right),\;a_{3}\left(i\right),\;\ldots ,\;a_{n}\left(i\right)\;\right]}^{T} \end{array}$$

and (${}^{T}$) denotes matrix transpose.

With the knowledge of $C_{i}$ and T, the nonlinear coefficients $a_{i}$ can be estimated by the LS method. The objective function is

(9) $$\arg\min_{a_{i}}{∥\mathrm{ones}\left(M,1\right)-C_{i}-T\cdot a_{i}∥}^{2}.$$

The least squares solution to Equation (8) is

(10) $${\hat{a}}_{i}={\left(T^{H}T\right)}^{-1}T^{H}\left(\mathrm{ones}\left(M,1\right)-C_{i}\right),$$

where ${\hat{a}}_{i}$ is the estimated nonlinear coefficients matrix.

## 4. Experimental Results and Discussion

**Figure 5:**
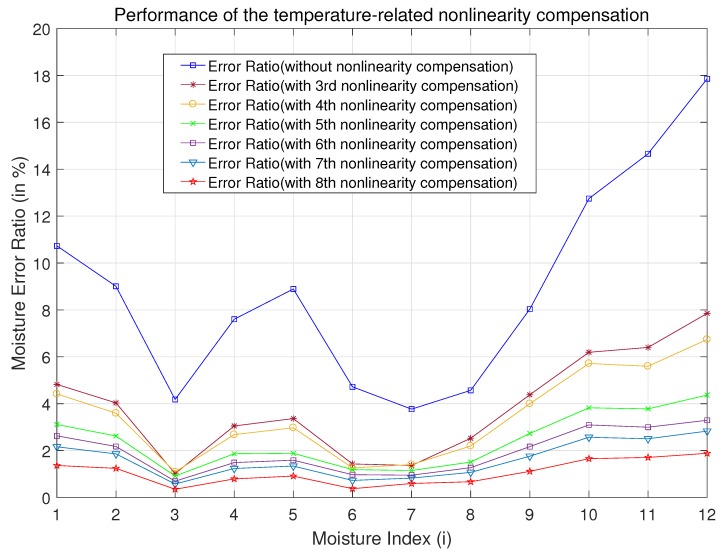
Performance validation of the temperature-related nonlinearity compensation(n = 3∼8).

Moisture error ratios under various nonlinear model-based compensation methods and the traditional methods are displayed in Figure 5. Measurement environments were kept the same throughout. It is obvious that the measurement error is very large without correction, which shows that temperature has a significant and nonlinear influence on the measurement results. In addition, measurement errors were greatly reduced when using third-order nonlinear correction. To further enhance sensor performance, we proposed and studied the adaptive order adjustable model. For the sensors studied in this article, the moisture error ratio reached a minimum under the eighth-order nonlinear compensation model, which indicated the best correction effect. Furthermore, corrections at even higher orders (ninth and above) would not yield better results as they would be overcorrected.

For instance, in the condition of moisture index i=1, the real moisture is $\theta_{real}\left(1\right)=40.3\%$, the sensing error ratio without nonlinearity compensation is as high as $R_{e,wo}\left(1\right)=10.73\%$; however, the error ratios with 3rd-order and 8th-order nonlinearity compensations are reduced to $R_{e,w}{\left(1\right)|}_{n=3}=4.822\%$ and $R_{e,w}{\left(1\right)|}_{n=8}=1.365\%$, respectively.

In the condition of moisture index i=12, the real moisture is $\theta_{real}\left(12\right)=12.6\%$, the measurement error ratio without nonlinearity compensation is $R_{e,wo}\left(12\right)=17.86\%$, but the error ratio with 3rd-order nonlinearity compensation is reduced to $R_{e,w}{\left(12\right)|}_{n=3}=7.852\%$, while the error ratio with 8th-order nonlinearity compensation is $R_{e,w}{\left(12\right)|}_{n=8}=1.885\%$. It is verified that the proposed adaptive nonlinear order regulating model-based nonlinearity compensation algorithm can obtain the optimal nonlinear order and achieve the best compensation performance.

To be clear, the 3∼8th-order nonlinear coefficients are estimated and listed in Table 3, Table 4, Table 5, Table 6, Table 7 and Table 8, respectively.

## 5. Conclusions

This paper presents an adaptive nonlinear order regulating model-based nonlinear compensation system for integrated temperature and moisture sensors. On the basis of a temperature-dependent multiorder nonlinear model for compensation correction, the measurement performance of each order nonlinear model and traditional method is compared. The experimental results verify that the nonlinear model compensation method can significantly reduce the sensing error, and the eighth order nonlinear model achieves the best measurement performance on the the integrated temperature and moisture sensor deployed in the wheatland in Zhejiang academy of agricultural sciences. Through this adaptive nonlinear compensation method, the measurement performance of the temperature and moisture sensor is greatly improved.

## Figures and Tables

**Table 1:** Comparison of three temperature and moisture sensors’ architectures.

Sensors’ Architectures	Linearization Technology	Advantages	Disadvantages
Conventional sensor (Figure 2)	LUT-based linearization	Simple and easy-to-achieve	Too many experiments, too much memory consumption, poor performance
Current sensor (Figure 3)	Model-based linearization	Practical and efficient	Inflexible, performance can be improved
Proposed sensor (Figure 4)	Adaptive nonlinear order regulating model-based linearization	Perfect performance	More computational resources

**Table 2:** Experimental data of temperature and moisture sensing (in %).

Temp. Idx. *m*	1	2	3	4	5	6	7	8	Real
**Temp.** $T_{m}$	**5 ${}^{\circ }$C**	**10 ${}^{\circ }$C**	**15 ${}^{\circ }$C**	**20 ${}^{\circ }$C**	**25 ${}^{\circ }$C**	**30 ${}^{\circ }$C**	**35 ${}^{\circ }$C**	**40 ${}^{\circ }$C**	**Moisture**
**Moisture Idx.** i	**Measured Moisture** $\theta_{\mathrm{meas}}\left(i,T_{m}\right)$								$\theta_{\mathrm{real}}\left(i\right)$
1	33.1	33.8	34.5	35.4	35.9	36.2	38.7	40.2	40.3
2	31.4	31.9	32.4	33.1	33.5	34.9	36.3	37.3	37.2
3	27.9	29.8	30.3	30.6	31.1	31.4	31.9	32.3	32.0
4	22.6	23.1	23.5	24.3	25.1	25.7	26.5	27.3	26.8
5	19.6	19.9	20.9	22.2	22.8	23.6	24.5	25.8	24.6
6	19.1	20.4	20.8	21.4	22.6	23.2	25.0	25.1	23.3
7	17.2	18.3	18.9	19.5	19.9	21.1	22.3	23.7	20.9
8	16.7	17.5	18.1	18.8	19.6	20.6	21.5	22.8	18.6
9	15.1	15.7	16.7	17.4	18.3	19.7	20.7	21.6	16.8
10	14.3	14.8	15.5	16.1	17.1	18.5	19.6	20.3	15.1
11	13.6	14.1	14.8	15.0	15.8	16.7	18.1	19.4	13.9
12	12.5	13.2	13.9	14.1	14.5	15.3	17.2	18.1	12.6

**Table 3:** Estimated nonlinear coefficients for the 3rd nonlinear order model.

Test No.	Estimated Nonlinear Coefficients		
i	$\;{\hat{a}}_{1}\left(i\right)\;$	$\;{\hat{a}}_{2}\left(i\right)\;$	$\;{\hat{a}}_{3}\left(i\right)\;$
1	2.3059 $\times \;10^{-3}$	−0.1388 $\times \;10^{-3}$	−0.0048 $\times \;10^{-3}$
2	2.3831 $\times \;10^{-3}$	−0.1185 $\times \;10^{-3}$	−0.0052 $\times \;10^{-3}$
3	0.6369 $\times \;10^{-3}$	−0.0511 $\times \;10^{-3}$	0.0007 $\times \;10^{-3}$
4	2.3190 $\times \;10^{-3}$	−0.1013 $\times \;10^{-3}$	−0.0043 $\times \;10^{-3}$
5	3.0301 $\times \;10^{-3}$	−0.1183 $\times \;10^{-3}$	−0.0045 $\times \;10^{-3}$
6	2.5375 $\times \;10^{-3}$	−0.0515 $\times \;10^{-3}$	−0.0019 $\times \;10^{-3}$
7	2.2943 $\times \;10^{-3}$	−0.0170 $\times \;10^{-3}$	0.0004 $\times \;10^{-3}$
8	1.8741 $\times \;10^{-3}$	0.0943 $\times \;10^{-3}$	0.0035 $\times \;10^{-3}$
9	2.3390 $\times \;10^{-3}$	0.1327 $\times \;10^{-3}$	0.0040 $\times \;10^{-3}$
10	2.2872 $\times \;10^{-3}$	0.1928 $\times \;10^{-3}$	0.0050 $\times \;10^{-3}$
11	1.5311 $\times \;10^{-3}$	0.2383 $\times \;10^{-3}$	0.0081 $\times \;10^{-3}$
12	1.0382 $\times \;10^{-3}$	0.2828 $\times \;10^{-3}$	0.0109 $\times \;10^{-3}$

**Table 4:** Estimated nonlinear coefficients for the 4th nonlinear order model.

Test No.	Estimated Nonlinear Coefficients			
i	$\;{\hat{a}}_{1}\left(i\right)\;$	$\;{\hat{a}}_{2}\left(i\right)\;$	$\;{\hat{a}}_{3}\left(i\right)\;$	$\;{\hat{a}}_{4}\left(i\right)\;$
1	4.0052 $\times \;10^{-4}$	−3.8107 $\times \;10^{-4}$	7.5852 $\times \;10^{-6}$	1.0460 $\times \;10^{-6}$
2	9.9438 $\times \;10^{-4}$	−2.9512 $\times \;10^{-4}$	3.7633 $\times \;10^{-6}$	7.6233 $\times \;10^{-7}$
3	4.0457 $\times \;10^{-4}$	−8.0658 $\times \;10^{-5}$	2.2654 $\times \;10^{-6}$	1.2754 $\times \;10^{-7}$
4	1.1753 $\times \;10^{-3}$	−2.4673 $\times \;10^{-4}$	3.0875 $\times \;10^{-6}$	6.2788 $\times \;10^{-7}$
5	1.4789 $\times \;10^{-3}$	−3.1559 $\times \;10^{-4}$	5.5129 $\times \;10^{-6}$	8.5151 $\times \;10^{-7}$
6	2.4366 $\times \;10^{-3}$	−6.4367 $\times \;10^{-5}$	−1.2525 $\times \;10^{-6}$	5.5407 $\times \;10^{-8}$
7	1.9509 $\times \;10^{-3}$	−6.0701 $\times \;10^{-5}$	2.6894 $\times \;10^{-6}$	1.8852 $\times \;10^{-7}$
8	2.7507 $\times \;10^{-3}$	2.0581 $\times \;10^{-4}$	−2.1841 $\times \;10^{-6}$	−4.8120 $\times \;10^{-7}$
9	3.9083 $\times \;10^{-3}$	3.3228 $\times \;10^{-4}$	−6.1429 $\times \;10^{-6}$	−8.6141 $\times \;10^{-7}$
10	4.5520 $\times \;10^{-3}$	4.8080 $\times \;10^{-4}$	−9.7061 $\times \;10^{-6}$	−1.2432 $\times \;10^{-6}$
11	3.7140 $\times \;10^{-3}$	5.1586 $\times \;10^{-4}$	−6.0784 $\times \;10^{-6}$	−1.1983 $\times \;10^{-6}$
12	3.8067 $\times \;10^{-3}$	6.3482 $\times \;10^{-4}$	−7.1164 $\times \;10^{-6}$	−1.5197 $\times \;10^{-6}$

**Table 5:** Estimated nonlinear coefficients for the 5th nonlinear order model.

Test No.	Estimated Nonlinear Coefficients				
i	$\;{\hat{a}}_{1}\left(i\right)\;$	$\;{\hat{a}}_{2}\left(i\right)\;$	$\;{\hat{a}}_{3}\left(i\right)\;$	$\;{\hat{a}}_{4}\left(i\right)\;$	$\;{\hat{a}}_{5}\left(i\right)\;$
1	2.258 $\times \;10^{-3}$	−5.365 $\times \;10^{-4}$	−1.939 $\times \;10^{-5}$	1.864 $\times \;10^{-6}$	7.825 $\times \;10^{-8}$
2	2.698 $\times \;10^{-3}$	−4.376 $\times \;10^{-4}$	−2.097 $\times \;10^{-5}$	1.512 $\times \;10^{-6}$	7.174 $\times \;10^{-8}$
3	1.214 $\times \;10^{-3}$	−1.483 $\times \;10^{-4}$	−9.480 $\times \;10^{-6}$	4.836 $\times \;10^{-7}$	3.407 $\times \;10^{-8}$
4	2.580 $\times \;10^{-3}$	−3.642 $\times \;10^{-4}$	−1.730 $\times \;10^{-5}$	1.246 $\times \;10^{-6}$	5.915 $\times \;10^{-8}$
5	2.562 $\times \;10^{-3}$	−4.062 $\times \;10^{-4}$	−1.021 $\times \;10^{-5}$	1.328 $\times \;10^{-6}$	4.562 $\times \;10^{-8}$
6	3.223 $\times \;10^{-3}$	−1.302 $\times \;10^{-4}$	−1.267 $\times \;10^{-5}$	4.016 $\times \;10^{-7}$	3.313 $\times \;10^{-8}$
7	2.672 $\times \;10^{-3}$	−1.211 $\times \;10^{-4}$	−7.783 $\times \;10^{-6}$	5.060 $\times \;10^{-7}$	3.038 $\times \;10^{-8}$
8	1.928 $\times \;10^{-3}$	2.746 $\times \;10^{-4}$	9.760 $\times \;10^{-6}$	−8.433 $\times \;10^{-7}$	−3.465 $\times \;10^{-8}$
9	2.044 $\times \;10^{-3}$	4.883 $\times \;10^{-4}$	2.093 $\times \;10^{-5}$	−1.682 $\times \;10^{-6}$	−7.853 $\times \;10^{-8}$
10	2.173 $\times \;10^{-3}$	6.799 $\times \;10^{-4}$	2.484 $\times \;10^{-5}$	−2.290 $\times \;10^{-6}$	−1.002 $\times \;10^{-7}$
11	1.189 $\times \;10^{-3}$	7.272 $\times \;10^{-4}$	3.059 $\times \;10^{-5}$	−2.310 $\times \;10^{-6}$	−1.064 $\times \;10^{-7}$
12	4.343 $\times \;10^{-4}$	9.170 $\times \;10^{-4}$	4.185 $\times \;10^{-5}$	−3.004 $\times \;10^{-6}$	−1.420 $\times \;10^{-7}$

**Table 6:** Estimated nonlinear coefficients for the 6th nonlinear order model.

Test No.	Estimated Nonlinear Coefficients					
i	$\;{\hat{a}}_{1}\left(i\right)\;$	$\;{\hat{a}}_{2}\left(i\right)\;$	$\;{\hat{a}}_{3}\left(i\right)\;$	$\;{\hat{a}}_{4}\left(i\right)\;$	$\;{\hat{a}}_{5}\left(i\right)\;$	$\;{\hat{a}}_{6}\left(i\right)\;$
1	−1.283 $\times \;10^{-3}$	−9.107 $\times \;10^{-4}$	5.032 $\times \;10^{-5}$	6.986 $\times \;10^{-6}$	−1.671 $\times \;10^{-7}$	−1.515 $\times \;10^{-8}$
2	3.948 $\times \;10^{-4}$	−6.810 $\times \;10^{-4}$	2.437 $\times \;10^{-5}$	4.843 $\times \;10^{-6}$	−8.783 $\times \;10^{-8}$	−9.853 $\times \;10^{-9}$
3	3.672 $\times \;10^{-4}$	−2.378 $\times \;10^{-4}$	7.181 $\times \;10^{-6}$	1.708 $\times \;10^{-6}$	−2.457 $\times \;10^{-8}$	−3.621 $\times \;10^{-9}$
4	7.791 $\times \;10^{-4}$	−5.545 $\times \;10^{-4}$	1.814 $\times \;10^{-5}$	3.851 $\times \;10^{-6}$	−6.562 $\times \;10^{-8}$	−7.704 $\times \;10^{-9}$
5	7.840 $\times \;10^{-4}$	−5.941 $\times \;10^{-4}$	2.479 $\times \;10^{-5}$	3.900 $\times \;10^{-6}$	−7.759 $\times \;10^{-8}$	−7.608 $\times \;10^{-9}$
6	1.487 $\times \;10^{-3}$	−3.137 $\times \;10^{-4}$	2.151 $\times \;10^{-5}$	2.914 $\times \;10^{-6}$	−8.720 $\times \;10^{-8}$	−7.430 $\times \;10^{-9}$
7	1.806 $\times \;10^{-3}$	−2.126 $\times \;10^{-4}$	9.270 $\times \;10^{-6}$	1.759 $\times \;10^{-6}$	−2.965 $\times \;10^{-8}$	−3.706 $\times \;10^{-9}$
8	3.228 $\times \;10^{-3}$	4.120 $\times \;10^{-4}$	−1.583 $\times \;10^{-5}$	−2.723 $\times \;10^{-6}$	5.542 $\times \;10^{-8}$	5.561 $\times \;10^{-9}$
9	4.596 $\times \;10^{-3}$	7.580 $\times \;10^{-4}$	−2.931 $\times \;10^{-5}$	−5.374 $\times \;10^{-6}$	9.831 $\times \;10^{-8}$	1.092 $\times \;10^{-8}$
10	5.370 $\times \;10^{-3}$	1.018 $\times \;10^{-3}$	−3.810 $\times \;10^{-5}$	−6.915 $\times \;10^{-6}$	1.213 $\times \;10^{-7}$	1.368 $\times \;10^{-8}$
11	4.205 $\times \;10^{-3}$	1.046 $\times \;10^{-3}$	−2.878 $\times \;10^{-5}$	−6.672 $\times \;10^{-6}$	1.026 $\times \;10^{-7}$	1.290 $\times \;10^{-8}$
12	3.272 $\times \;10^{-3}$	1.217 $\times \;10^{-3}$	−1.401 $\times \;10^{-5}$	−7.108 $\times \;10^{-6}$	5.458 $\times \;10^{-8}$	1.214 $\times \;10^{-8}$

**Table 7:** Estimated nonlinear coefficients for the 7th nonlinear order model.

Test No.	Estimated Nonlinear Coefficients						
i	$\;{\hat{a}}_{1}\left(i\right)\;$	$\;{\hat{a}}_{2}\left(i\right)\;$	$\;{\hat{a}}_{3}\left(i\right)\;$	$\;{\hat{a}}_{4}\left(i\right)\;$	$\;{\hat{a}}_{5}\left(i\right)\;$	$\;{\hat{a}}_{6}\left(i\right)\;$	$\;{\hat{a}}_{7}\left(i\right)\;$
1	1.921 $\times \;10^{-3}$	−1.904 $\times \;10^{-3}$	−7.442 $\times \;10^{-5}$	2.279 $\times \;10^{-5}$	1.036 $\times \;10^{-6}$	−6.598 $\times \;10^{-8}$	−3.154 $\times \;10^{-9}$
2	2.820 $\times \;10^{-3}$	−1.433 $\times \;10^{-3}$	−7.006 $\times \;10^{-5}$	1.681 $\times \;10^{-5}$	8.229 $\times \;10^{-7}$	−4.833 $\times \;10^{-8}$	−2.388 $\times \;10^{-9}$
3	1.210 $\times \;10^{-3}$	−4.993 $\times \;10^{-4}$	−2.564 $\times \;10^{-5}$	5.868 $\times \;10^{-6}$	2.920 $\times \;10^{-7}$	−1.700 $\times \;10^{-8}$	−8.300 $\times \;10^{-10}$
4	2.482 $\times \;10^{-3}$	−1.083 $\times \;10^{-3}$	−4.816 $\times \;10^{-5}$	1.225 $\times \;10^{-5}$	5.739 $\times \;10^{-7}$	−3.472 $\times \;10^{-8}$	−1.677 $\times \;10^{-9}$
5	2.409 $\times \;10^{-3}$	−1.098 $\times \;10^{-3}$	−3.850 $\times \;10^{-5}$	1.192 $\times \;10^{-5}$	5.328 $\times \;10^{-7}$	−3.339 $\times \;10^{-8}$	−1.600 $\times \;10^{-9}$
6	2.821 $\times \;10^{-3}$	−7.277 $\times \;10^{-4}$	−3.045 $\times \;10^{-5}$	9.499 $\times \;10^{-6}$	4.140 $\times \;10^{-7}$	−2.860 $\times \;10^{-8}$	−1.314 $\times \;10^{-9}$
7	2.661 $\times \;10^{-3}$	−4.778 $\times \;10^{-4}$	−2.402 $\times \;10^{-5}$	5.977 $\times \;10^{-6}$	2.914 $\times \;10^{-7}$	−1.727 $\times \;10^{-8}$	−8.417 $\times \;10^{-10}$
8	1.823 $\times \;10^{-3}$	8.478 $\times \;10^{-4}$	3.888 $\times \;10^{-5}$	−9.656 $\times \;10^{-6}$	−4.722 $\times \;10^{-7}$	2.785 $\times \;10^{-8}$	1.383 $\times \;10^{-9}$
9	2.382 $\times \;10^{-3}$	1.445 $\times \;10^{-3}$	5.690 $\times \;10^{-5}$	−1.630 $\times \;10^{-5}$	−7.332 $\times \;10^{-7}$	4.605 $\times \;10^{-8}$	2.180 $\times \;10^{-9}$
10	2.304 $\times \;10^{-3}$	1.969 $\times \;10^{-3}$	8.126 $\times \;10^{-5}$	−2.204 $\times \;10^{-5}$	−1.030 $\times \;10^{-6}$	6.231 $\times \;10^{-8}$	3.018 $\times \;10^{-9}$
11	1.513 $\times \;10^{-3}$	1.881 $\times \;10^{-3}$	7.601 $\times \;10^{-5}$	−1.995 $\times \;10^{-5}$	−9.081 $\times \;10^{-7}$	5.560 $\times \;10^{-8}$	2.650 $\times \;10^{-9}$
12	1.159 $\times \;10^{-4}$	2.196 $\times \;10^{-3}$	1.089 $\times \;10^{-4}$	−2.268 $\times \;10^{-5}$	−1.131 $\times \;10^{-6}$	6.221 $\times \;10^{-8}$	3.107 $\times \;10^{-9}$

**Table 8:** Estimated nonlinear coefficients for the 8th nonlinear order model.

Test No.	Estimated Nonlinear Coefficients							
i	$\;{\hat{a}}_{1}\left(i\right)\;$	$\;{\hat{a}}_{2}\left(i\right)\;$	$\;{\hat{a}}_{3}\left(i\right)\;$	$\;{\hat{a}}_{4}\left(i\right)\;$	$\;{\hat{a}}_{5}\left(i\right)\;$	$\;{\hat{a}}_{6}\left(i\right)\;$	$\;{\hat{a}}_{7}\left(i\right)\;$	$\;{\hat{a}}_{8}\left(i\right)\;$
1	−9.155 $\times \;10^{-4}$	3.239 $\times \;10^{-2}$	−3.458 $\times \;10^{-2}$	8.583 $\times \;10^{-3}$	−1.815 $\times \;10^{-3}$	1.067 $\times \;10^{-4}$	−5.813 $\times \;10^{-6}$	−6.912 $\times \;10^{-10}$
2	7.324 $\times \;10^{-4}$	2.979 $\times \;10^{-2}$	−2.923 $\times \;10^{-2}$	7.514 $\times \;10^{-3}$	−1.548 $\times \;10^{-3}$	9.244 $\times \;10^{-5}$	−4.967 $\times \;10^{-6}$	−5.057 $\times \;10^{-10}$
3	2.747 $\times \;10^{-4}$	1.939 $\times \;10^{-2}$	−1.799 $\times \;10^{-2}$	5.398 $\times \;10^{-3}$	−1.107 $\times \;10^{-3}$	7.096 $\times \;10^{-5}$	−3.808 $\times \;10^{-6}$	−1.637 $\times \;10^{-10}$
4	9.766 $\times \;10^{-4}$	3.094 $\times \;10^{-2}$	−2.723 $\times \;10^{-2}$	7.688 $\times \;10^{-3}$	−1.496 $\times \;10^{-3}$	9.426 $\times \;10^{-5}$	−4.884 $\times \;10^{-6}$	−3.529 $\times \;10^{-10}$
5	7.935 $\times \;10^{-4}$	4.380 $\times \;10^{-2}$	−3.524 $\times \;10^{-2}$	1.083 $\times \;10^{-2}$	−1.985 $\times \;10^{-3}$	1.311 $\times \;10^{-4}$	−6.516 $\times \;10^{-6}$	−3.492 $\times \;10^{-10}$
6	1.526 $\times \;10^{-3}$	3.937 $\times \;10^{-2}$	−2.354 $\times \;10^{-2}$	9.513 $\times \;10^{-3}$	−1.538 $\times \;10^{-3}$	1.159 $\times \;10^{-4}$	−5.435 $\times \;10^{-6}$	−2.947 $\times \;10^{-10}$
7	1.526 $\times \;10^{-3}$	4.150 $\times \;10^{-2}$	−2.016 $\times \;10^{-2}$	1.006 $\times \;10^{-2}$	−1.410 $\times \;10^{-3}$	1.218 $\times \;10^{-4}$	−5.181 $\times \;10^{-6}$	−1.637 $\times \;10^{-10}$
8	2.838 $\times \;10^{-3}$	3.561 $\times \;10^{-2}$	3.262 $\times \;10^{-4}$	7.713 $\times \;10^{-3}$	−4.711 $\times \;10^{-4}$	8.782 $\times \;10^{-5}$	−2.434 $\times \;10^{-6}$	2.947 $\times \;10^{-10}$
9	3.815 $\times \;10^{-3}$	4.165 $\times \;10^{-2}$	4.732 $\times \;10^{-3}$	8.636 $\times \;10^{-3}$	−3.747 $\times \;10^{-4}$	9.545 $\times \;10^{-5}$	−2.290 $\times \;10^{-6}$	4.929 $\times \;10^{-10}$
10	4.639 $\times \;10^{-3}$	3.822 $\times \;10^{-2}$	1.646 $\times \;10^{-2}$	7.210 $\times \;10^{-3}$	1.778 $\times \;10^{-4}$	7.484 $\times \;10^{-5}$	−6.800 $\times \;10^{-7}$	6.567 $\times \;10^{-10}$
11	3.399 $\times \;10^{-3}$	3.327 $\times \;10^{-2}$	2.385 $\times \;10^{-2}$	6.242 $\times \;10^{-3}$	5.424 $\times \;10^{-4}$	6.297 $\times \;10^{-5}$	3.400 $\times \;10^{-7}$	5.823 $\times \;10^{-10}$
12	2.319 $\times \;10^{-3}$	3.188 $\times \;10^{-2}$	3.014 $\times \;10^{-2}$	5.931 $\times \;10^{-3}$	7.631 $\times \;10^{-4}$	5.879 $\times \;10^{-5}$	8.521 $\times \;10^{-7}$	6.412 $\times \;10^{-10}$
